# Tubular glassy carbon microneedles with fullerene-like tips for biomedical applications

**DOI:** 10.3762/bjnano.13.38

**Published:** 2022-05-19

**Authors:** Sharali Malik, George E Kostakis

**Affiliations:** 1 Institute of Quantum Materials and Technology (IQMT), Karlsruhe Institute of Technology (KIT), Hermann-von-Helmholtz-Platz 1, 76344 Eggenstein-Leopoldshafen, Germany; 2 Chemistry Department, School of Life Sciences, University of Sussex, Falmer, BN1 9QG, UK

**Keywords:** biomedical, glassy carbon, microneedle, neural engineering, COST Action EsSENce CA19118

## Abstract

Glassy carbon, in general, is made by the pyrolysis of polymeric materials and has been the subject of research for at least fifty years. However, as understanding its microstructure is far from straightforward, it continues to be an area of active research. Glassy carbon adopts different allotropes depending on the hybridizations of the C–C bond, that is, sp, sp^2^, or sp^3^*.* Furthermore, a variety of short-range ordering effects can interact with each other and this, along with the effects of microporosity, grain boundaries, and defects, render this a fascinating material. Following the nanoarchitectonics concept of bottom-up creation of functional materials, we use methane rather than a polymer to form glassy carbon. Here we show that tubular glassy carbon microneedles with fullerene-like tips form when methane undergoes pyrolysis on a curved alumina surface. X-ray diffraction of these glassy carbon tubules shows long-range order with a *d*-spacing of 4.89 Å, which is indicative of glassy carbon. Raman spectroscopy shows the material to be graphitic in nature, and SEM shows the fullerene-like structure of the material. This work provides new insights into the structure of glassy carbons relevant to the application of glassy carbons as a biomaterial, for example, as a new form of carbon-based microneedles. Since metallic needles can introduce toxic/allergenic species into susceptible subjects, this alternative carbon-based microneedle form has great potential as a replacement biomedical material for metallic needles in the field of neural engineering and as acupuncture needles.

## Introduction

Glassy carbon, also known as “glass-like carbon” or “vitreous carbon” is an allotrope of carbon, which combines glassy and ceramic properties with those associated with graphite and has been of scientific and technological interest for over fifty years. Glassy carbon has good electrical and thermal conductivities, excellent chemical stability, and good biocompatibility, which has led to many advanced technological applications [1]. The use of glassy carbon as an electrode material in electrochemistry is probably its best-known application. However, understanding the microstructure of glassy carbon is far from straightforward, therefore, this continues to be an area of active research.

R. E. Franklin, best known for her poorly acknowledged role in discovering the structure of DNA, formulated the first structural models for what is now known as glassy carbon and coined the terms “graphitizing” and “non-graphitizing carbons” to describe these materials in the 1940s [2–3]. B. Redfern [4] first used the term “vitreous carbon” in the 1950s when he saw its first technological application as a crucible material. The term “glassy carbon” was used for the first time in 1962, by S. Yamada and H. Sato [5] in their research on the physical properties of carbon materials. Although the IUPAC recommends the use of the term glass-like carbon [6], the scientific world has become used to the term glassy carbon (GC). It is also worth noting that the term glassy carbon or glass-like carbon is not used in some articles in which the materials have properties of glassy carbon. In this article, the authors use the term glassy carbon in order to ensure its wider dissemination.

Franklin stated that “the structure of carbons formed by pyrolysis of organic materials depends not only on the temperature of preparation, but also, to a very large extent, on the nature of the starting material” [2]. In the 1970s, G. M. Jenkins and K. Kawamura examined the structure of glassy carbon [7]. They noted, “the lack of information about the starting materials and the carbonization process given by manufacturers precludes the elucidation of the structure of the material; it is necessary to study carbonization mechanisms in well-defined starting materials.” E. E. Hucke and his team in their 1972 report on glassy carbons [8] state, “from data previously available in the literature and the early results of this program, it has become obvious that “glassy carbon” is not a single material, since even though it contains essentially only carbon atoms, its structure can be varied at all size levels.” More recently, S. Sharma in a review of glassy carbon materials concluded that “it has been confirmed by both theoretical and experimental investigation that the chemical composition of the polymer plays the most important role in determining the nature of the resulting glassy carbon” [9]. Sharma has also coined the term “polymer-derived carbons” for glassy carbon made by the thermal decomposition of polymers, which accounts for virtually all the glassy carbons found in the literature to date.

In this article, we have followed the nanoarchitectonics [10] concept to fabricate our glassy carbon material by using methane as building unit and carbon source rather than polymers. There are earlier works on methane pyrolysis in a flow reactor by F. G. Billaud et al. [11] and Z. Bai and co-workers [12]. Bai et al. observed that carbon deposition in the mesopores of alumina is responsible for catalytic activity resulting in the decomposition of methane [12].

Here, we examine the characteristics of the glassy carbon produced by catalytic pyrolysis of methane. Our results show clear experimental evidence for a fullerene-like structure. So far, evidence for this was inferred from TEM observations and theoretical models of polymer-derived carbons [13]. Since glassy carbon has been identified as a promising material for electrodes used in neural prosthetics [1], our long-term aim is to develop a scaleable procedure utilising catalytic methane pyrolysis to fabricate glassy carbon microneedle electrodes for biomedical applications.

## Results and Discussion

### Growth of glassy carbon microneedles

Previously, glassy carbon microneedles have been made by the pyrolysis of commercially available polymers. The polymer material was pre-patterned as an array, which was then converted to glassy carbon microneedle patches via a conventional carbon-microelectromechanical system (C-MEMS) process [14]. In this article we report the fabrication of freestanding glassy carbon microneedles in a single step achieved by the pyrolysis of methane on a curved alumina surface. The surface provides the catalyst as well as the “strain” required to direct nucleation and growth.

**Figure 1:**
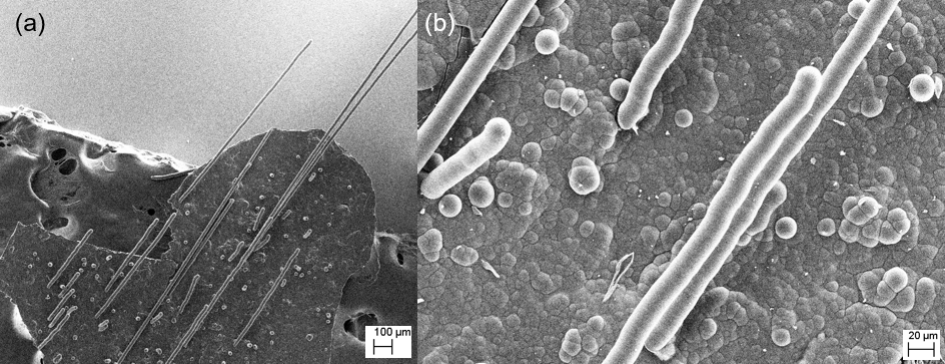
(a) SEM overview image showing a number of glassy carbon microneedles, (b) SEM detail image of glassy carbon microneedles, nucleating microneedles, and nucleation “blisters”.

Figure 1a is a scanning electron microscopy (SEM) overview image showing a number of glassy carbon microneedles, which grow in the direction of the gas flow. Figure 1b is a SEM detail image showing glassy carbon microneedles, nucleating microneedles, and “blisters”, which correspond to the early stages of the microneedle growth. Figure 1 shows that the microneedles grown under the given pyrolysis conditions are uniformly ca. 25 µm in diameter.

**Figure 2:**
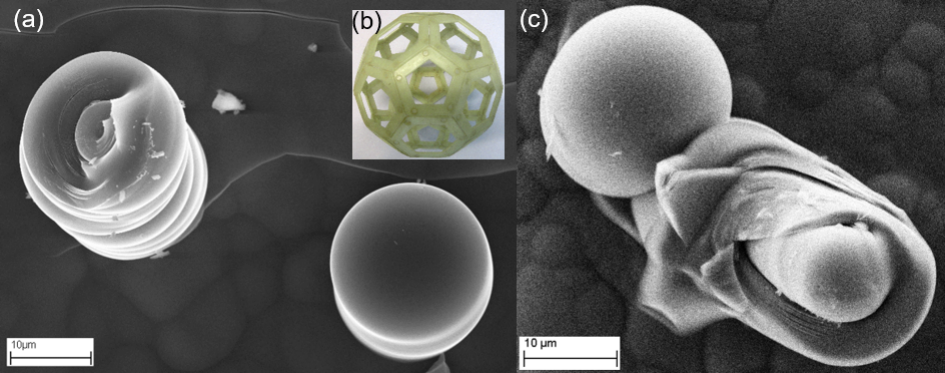
(a) SEM overview image of two glassy carbon tubules; the one on the right has its fullerene dome intact and the one on the left has a fractured fullerene dome giving insights into the internal concentric structure; (b) inset of a model of a fullerene; (c) SEM overview image of two glassy carbon tubules; the one on the left has its dome intact and the one on the right is fractured showing some of the inner intact fullerene domes.

The fullerene-like tips of the microneedles can clearly be seen in the SEM micrographs in Figure 2a and Figure 2c. Figure 2b shows a model of a fullerene, which highlights the requirement of pentagons as well as hexagons in order to close the microneedle tips.

SEM-EDX measurements (EDX INCA 400, Oxford Instruments, at 20 kV) showed that the materials are only carbon with no other detectable elements. The rounded caps of the glassy carbon tubules suggest that they have characteristics of fullerenes regarding the need for pentagons in addition to hexagons to close the cap [15] (see the model of fullerene C_60_ in Figure 2b). Therefore, we consider the most likely growth mechanism to be that proposed by S. Amelinckx and co-workers [16]. Their model explains the formation of multishell closure domes in which nucleation is attributed to the initial formation of fullerene domes. These originate from the “blisters” observed in Figure 1b near the glassy carbon microneedles.

**Figure 3:**
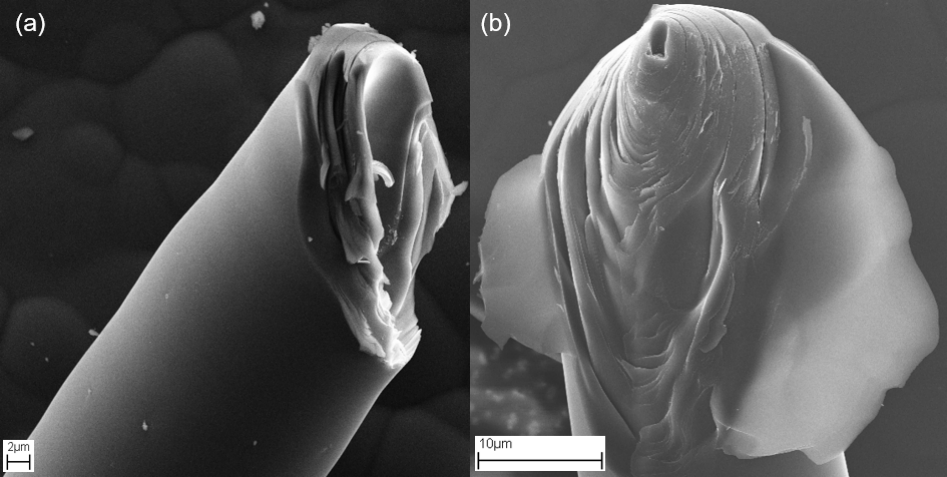
SEM images of two typical glassy carbon microneedles fractured at the tip showing that the microneedles have a hollow core and are tubular in structure.

The SEM images in Figure 3 show two typical glassy carbon microneedles fractured at the tip revealing their internal structure. This is clear experimental evidence that the glassy carbon microneedles are hollow in nature.

### Characterization of the glassy carbon microneedles

**Figure 4:**
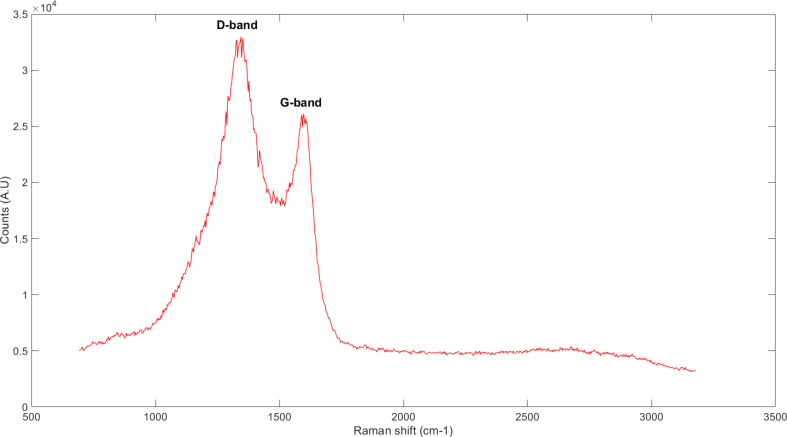
Raman spectrum of glassy carbon tubules. Both D-band and G-band are sharp and well defined, which indicates graphitic rather than amorphous carbon [19].

Figure 4 shows a typical Raman spectrum of the glassy carbon microneedles. The D-band is at 1352 cm^−1^, and the G-band is at 1589 cm^−1^. The D-band, the so-called defect band, originates from a hybridized vibrational mode associated with local defects and disorder. In this case, it results from the curvature of the glassy carbon tubules. The G-band, the so-called graphitic or tangential band, is characteristic of graphite and originates from the in-plane tangential stretching of the C–C bonds. The intensity of the D-band is much higher than that of the G-band, showing the local crystalline structure [17]. This is in good agreement with Raman spectrum data for glassy carbon [17–18] and confirms that the carbon microneedles fabricated here are glassy in nature.

**Figure 5:**
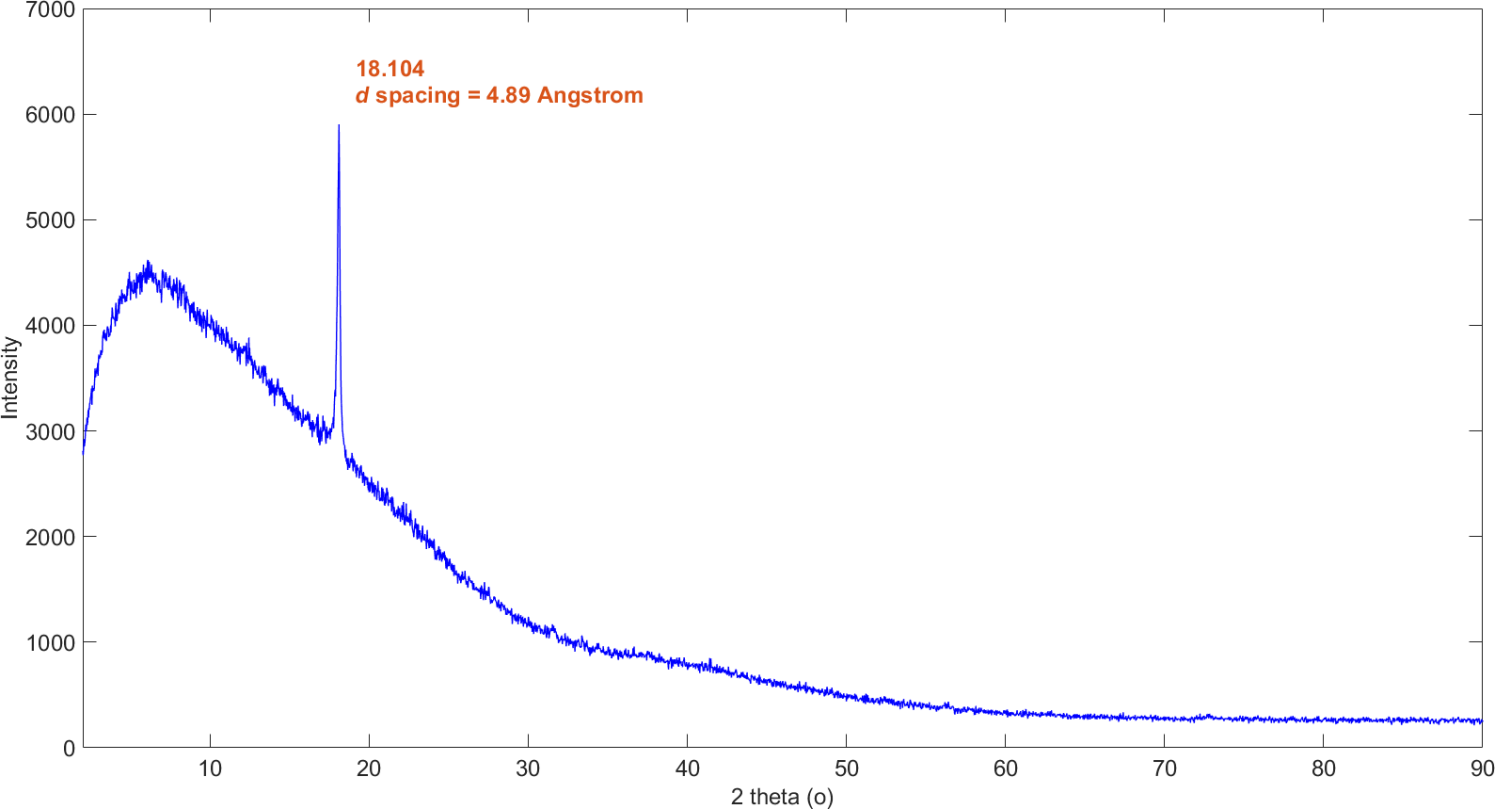
XRD of glassy carbon tubules, including the calculation of the interlayer spacing.

Figure 5 shows the XRD measurement of the glassy carbon tubules. The single sharp peak is indicative of graphitic carbon with long-range crystalline order. The interlayer spacing is calculated to have a *d*-spacing of 4.89 Å. Table 1 shows the interlayer spacing of graphite and other selected carbon materials and is further evidence that the tubules are glassy carbon.

In Table 1, the interlayer spacing data characteristic for selected carbon materials is shown. Graphitic carbon is known to have an interlayer spacing of 3.354 Å [20–21]. Glassy carbon has a much larger interlayer spacing than turbostratic carbon [22] and carbon fibres [23]. The interlayer spacing of the glassy carbon microneedles in this work is calculated from the XRD data to have a *d*-spacing of 4.89 Å, which is further experimental evidence that the microneedles are of glassy carbon.

**Table 1:** Interlayer spacing data characteristic for selected carbon materials.

Carbon material	*d*-spacing (Å)	Reference

graphite	3.354	[20–21]
turbostratic carbon	3.44–3.67	[22]
carbon fibre	3.40–3.53	[23]
glassy carbon (polymer-derived carbon)	3.45–3.60 3.46–3.70	[24–25]
glassy carbon (H^+^ irradiated graphite whiskers)	4.7–6.9	[26]
glassy carbon (CH_4_ derived carbon)	4.89	this work

Most glassy carbons prepared to date have been made by pyrolysis of polymeric materials. These generally do not have long-range crystalline order and undergo glass-like fracture. However, more recently, it has been shown that glassy carbons can graphitize under high-stress conditions [27–28]. Furthermore, it has been shown that intercalated species can increase the interlayer spacing of graphitic carbons [29]. It has also been shown that this increase in interlayer spacing in glassy carbon corresponds to the nature of the intercalating species [26]. Here the “stress” arises from the constraints of the growing glassy carbon layers from the curved alumina gas-flow tube and the intercalating species, which are gaseous species, generated when methane undergoes pyrolysis. These are transitory species and do not remain in the glassy carbon tubules. The early stages of this process can be seen, in Figure 1b, as the nucleation “blisters” on the surface of the glassy carbon formed on the alumina. These then develop into glassy carbon microneedles.

## Conclusion

In this work, we have shown that the pyrolysis of methane leads to the formation of glassy carbon microneedles. These were characterized and identified using a combination of SEM, Raman spectroscopy, and XRD. This simple method of preparation provides an easy and efficient alternative to previously used polymer pyrolysis reactions and provides a clean and effective means to access glassy carbon materials. Further work is planned to determine the pyrolysis conditions required for varying the diameter and length of the microneedles. This will be carried out in conjunction with more detailed characterization techniques such as XPS and TEM.

Recently, V. Uskoković [30] has pointed out that although glassy carbons have seen numerous application in the last fifty years, biomedical applications have been sporadic. I. Schreiver et al. [31] have shown that allergic reactions can be triggered by nickel and chrome particles released from surgical stainless steel needles during needle wear in human skin. Allergic reactions such as contact dermatitis resulting from the presence of nickel and chromium in acupuncture needles have also been reported [32]. Glassy carbon is biocompatible, electrically and thermally conducting, and mechanically strong [1]. Therefore, biomedical applications for glassy carbon microneedles include the use as alternatives to stainless steel surgical needles in acupuncture and as microelectrodes in neural prosthetics [9,14].

## Experimental

### Sample preparation

The samples were prepared by flowing methane (CH_4_) at a rate of 40 sccm for 12 h at *p* = 200 mbar and a gas temperature of 1020 °C through a 20 mm diameter alumina (Al_2_O_3_) tube (15 mm internal diameter), which was then cooled under argon (Ar).

### Characterization

The prepared glassy carbon microneedles were characterized by Raman spectroscopy (WiTec CRM200, laser excitation at 632.8 nm), scanning electron microscopy (SEM Leo 1530, with a spatial resolution of 1 nm at 20 kV and 3 nm at 1 kV, equipped with an energy-dispersive X-ray analysis system EDX INCA 400 from Oxford Instruments), and X-ray diffraction (STOE STADI-P diffractometer with Cu Kα radiation (λ = 1.5406 Å).
